# Considerations for transoral robotic surgery with fluorescence imaging: a narrative review

**DOI:** 10.1007/s11701-026-03724-8

**Published:** 2026-07-27

**Authors:** Akhilesh M. Wodeyar, Benjamin B. Kasten, Sherin James, Harishanker Jeyarajan, Anne C. Kane, Eben L. Rosenthal, Jason M. Warram

**Affiliations:** 1 Department of Otolaryngology, University of Alabama at Birmingham, Volker Hall G082, 1670 University Boulevard, Birmingham, AL 35294 USA; 2 Department of Otolaryngology-Head and Neck Surgery, Vanderbilt University, Nashville, TN USA

## Abstract

To review evidence and practical considerations for integrating fluorescence imaging into robotic head and neck surgery, emphasizing technical parameters, optical limitations, and clinical utility. We additionally incorporate insights derived from our institutional experience to contextualize real-world implementation challenges. Transoral robotic surgery (TORS) has expanded minimally invasive treatment for head and neck squamous cell carcinoma, improving functional outcomes and reducing morbidity. However, absent tactile feedback and limited depth perception remain challenges, particularly during margin assessment. Fluorescence-guided surgery (FGS) has emerged as a potential solution for real-time tumor visualization. This narrative review synthesizes literature on nonspecific dyes (indocyanine green) and tumor-targeted agents (panitumumab-IRDye800CW), evaluating compatibility with the da Vinci Firefly system. Institutional intraoperative observations complement existing data and highlight workflow considerations. Fluorescence imaging improves intraoperative tumor delineation and deep margin assessment. Critical technical variables include imaging mode selection, scope angle, illumination characteristics, field uniformity, and autoscaling behavior. Tumor-targeted tracers demonstrate superior contrast in Sensitive Firefly mode but require standardized protocols for optimal performance. Fluorescence-guided robotic surgery can improve oncologic precision in TORS by compensating for inherent sensory limitations. Consistent clinical benefit requires further development of targeted agents, workflow standardization, and robust validation studies.

**Supplementary Information:**

The online version contains supplementary material available at 10.1007/s11701-026-03724-8.

## Introduction

Transoral robotic surgery (TORS**)** has rapidly transitioned from early feasibility to standard practice across major head-and-neck centers worldwide, with adoption now spanning academic and high-volume community programs. Robotic abilities like magnified 3D visualization, tremor filtration, and wristed instrumentation enable precise transoral resections while preserving function [1]. This has led to widespread use and expansion of TORS for appropriately selected oropharyngeal and supraglottic tumors [2]. With most contemporary head and neck units now offering TORS and multi-institutional cohorts consistently reporting high negative-margin rates, low tracheostomy/gastrostomy dependence, and favorable locoregional control [3–5], the field has moved from “Can we do it?’’ to “How do we do it well and reproduce it safely everywhere?”

Despite the advances, robotic surgery introduces a fundamental challenge: the loss of tactile feedback and reduced ability to gauge tissue resistance, tumor firmness, and subtle anatomic transitions. In conventional open surgery, these sensory cues are critical for identifying tumor planes, assessing deep margins, and distinguishing tumor from inflamed or fibrotic tissue. Their absence in TORS contributes to uncertainty during dissection, especially in deep and poorly visualized regions, and increases the risk of positive or close margins. As patient selection has broadened and surgeons have pushed the limits of minimally invasive resection, the need for reliable intraoperative adjuncts to compensate for these sensory limitations has become increasingly evident.

Fluorescence-guided surgery (FGS) has emerged as a promising solution to this problem. Large body of evidence indicates that near-infrared (NIR) fluorescence imaging can significantly enhance surgical precision [6]. NIR fluorophores, both nonspecific vascular tracers and tumor-targeted agents, enable real-time visualization of tumor tissue, critical structures, perfusion patterns, and lymphatic pathways. Gowrishankar et al. highlighted that FGS is particularly valuable for addressing challenging deep margins, which contribute to most positive margins in head and neck cancer (HNC) [7]. In robotic surgery, fluorescence can restore a surrogate for lost tactile feedback by providing contrast-based cues that enhance the surgeon’s perception of tissue boundaries. Early clinical studies demonstrate the ability of fluorescence imaging to improve margin visualization, identify occult disease, and guide sentinel lymph node biopsy. Importantly, the integration of fluorescence into the da Vinci Firefly imaging system provides a practical, accessible platform for its application during TORS.

However, the effective use of fluorescence during robotic surgery requires more than simply activating a fluorescence mode. The interpretation of the NIR signal during surgery is profoundly influenced by optical physics, instrument design, imaging-system characteristics, fluorophore properties, and procedural workflow. Factors such as scope angle, working distance, autoscaling behavior, illumination spectra, and tissue scattering can alter perceived intensity and lead to misinterpretation if not understood. These constraints are especially relevant in the confined anatomy of the oropharynx and larynx, where small changes in scope orientation or tissue exposure can dramatically affect the fluorescence signal. Despite the growing enthusiasm for FGS, detailed guidance on how to apply these principles specifically within robotic head and neck surgery remains limited.

This review addresses that gap by synthesizing current evidence on fluorescence-guided robotic surgery in HNC and contextualizing it with technical and experiential insights from our own institutional use of targeted fluorescence imaging during TORS. We compare the performance of both nonspecific and tumor-targeted fluorophores, outline the capabilities and limitations of the Firefly imaging system, and highlight practical considerations that directly impact intraoperative interpretation. We also focus on the next steps on standardizing tumor targeted fluorescence guidance in TORS workflows so centers can achieve consistent oncologic precision as TORS continues its broad clinical diffusion. Our aim is to provide surgeons and researchers with a framework to understand the optical, technical, and clinical factors that govern fluorescence use in TORS, and to define the opportunities and challenges that will shape its future integration into HNC robotic surgery.

## Methods

We reviewed peer-reviewed literature indexed in PubMed, Embase, and Web of Science database from December 2009 (FDA clearance of da Vinci for the use of TORS in HNC) through March 2026 with additional sources identified by reference screening of key articles and device manuals. The search focused on publications related to TORS, NIR fluorescence imaging, nonspecific dyes such as indocyanine green (ICG), tumor-targeted fluorophores including anti-epidermal growth factor receptor (EGFR) agents, and the technical specifications of the da Vinci Firefly imaging system. To complement published data, we incorporated experiential observations from our ongoing use of fluorescence imaging during TORS procedures at our institution. These anecdotal observations are presented descriptively and used to highlight practical nuances that are under-represented in the literature.

### Fluorescent imaging in robotic head and neck surgery: current evidence

#### Nonspecific agent (ICG)

ICG is the most widely adopted intraoperative NIR fluorophore because of its favorable safety profile, rapid pharmacokinetics, and compatibility with NIR surgical cameras. ICG has since shown great potential during fluorescence-guided robotic surgeries in almost all specialties [8]. Early studies in HNC with ICG characterized its optical properties, administration techniques, and timing strategies [9], but subsequent clinical evaluation in TORS demonstrated limited oncologic utility, as ICG produces diffuse mucosal uptake without tumor-specific contrast. In a prospective series of six oropharyngeal squamous cell carcinoma (OPSCC) patients, the Firefly system failed to identify gross tumors, positive margins, vascular structures, or unknown primaries despite adequate mucosal fluorescence [10]. De Ravin et al. reported a pooled sensitivity of 91.7% and specificity of 71.9% using ICG fluorescence in their systematic review paper, with an average tumor-to-background ratio (TBR) of 1.56 [11]. This highlights high sensitivity but modest specificity for distinguishing tumors from surrounding tissues with a low TBR as compared with current targeted agents, reflecting its nonspecific vascular distribution.

Importantly, these limitations are related to tumor delineation and margin assessment in FGS-HNC surgeries, especially in inflamed or fibrotic tissues typical of head and neck squamous cell carcinoma (HNSCC), rather than to the broader intraoperative value of ICG. ICG fluorescence has been shown to be useful in TORS for perfusion and vascular permeability assessment [12], carcinoma of unknown primary [13], lymphatic mapping and sentinel lymph node (SLN) applications [14], parapharyngeal space navigation [15], thyroid and parathyroid surgery [16], and selected non-margin-based applications (Table 1), underscoring its role as an adjunctive physiologic and anatomic tracer rather than a tumor-specific oncologic agent. As fluorescence guidance has expanded, international Delphi-style consensus has highlighted persistent variability in technique and emphasized the need to standardize parameters such as dosing, timing, and imaging settings to improve reproducibility and interpretation [17]. 

#### Tumor-targeted agents (anti-EGFR conjugates and others)

Tumor-targeted probes, most prominently anti-EGFR monoclonal antibodies conjugated to NIR fluorophores (e.g., cetuximab-IRDye800CW and panitumumab-IRDye800CW), have emerged as leading agents for fluorescence guidance in HNSCC, leveraging the high prevalence of EGFR overexpression to improve tumor contrast relative to surrounding tissues. Early translational frameworks supporting repurposing clinically approved antibodies for imaging emphasized their favorable safety knowledge base and predictable pharmacokinetics, enabling efficient clinical translation compared with de novo tracers [18]. Broader field guidance also underscores that successful translation requires alignment among tracer performance, imaging-system characteristics, and standardized workflows and reporting practices rather than tracer properties alone [19]. Zhang et al. argue that advances in targeted NIR fluorophores, cancer biomarkers, and intraoperative detection hardware have enabled highly specific, real-time cancer visualization “beyond the margins,” while emphasizing that effective clinical use still depends on overcoming practical implementation challenges [20]. These issues are especially relevant in TORS, where targeted agents frequently generate lower absolute intensity than does ICG but provide higher specificity (Table 1); accordingly, accurate interpretation becomes disproportionately dependent on procedural imaging variables, making standardized protocols and documented settings essential for consistent margin interrogation.

Subsequent dose escalation work using panitumumab-IRDye800CW showed that tumor-targeted fluorescence can support intraoperative decision-making not only through in situ visualization but also through ex vivo specimen mapping, where fluorescence contrast is often greater than in the operative field. NIR fluorescence in tissues correlated with histology and improved assessment of tumor proximity to cut specimen surfaces [21]. Together, these findings open a complementary ex vivo diagnostic workflow leveraging rapid, histology-correlated fluorescence to triage and localize close/positive margin risk immediately after resection. In the interim phase II cohort of OPSCC patients undergoing TORS, panitumumab-IRDye800CW produced consistent tumor-specific fluorescence on the integrated da Vinci Xi camera (mean intraoperative TBR ~ 10.7), correlated with histopathology, and even enabled detection of an occult residual tumor fragment not appreciated on brightfield imaging [22]. These findings support a practical *intraoperative* workflow in TORS incorporating tumor-targeted real-time fluorescence interrogation (including a post-resection cavity scan) to detect tumor margins and occult residual disease and to guide margin-directed decision-making when tactile feedback is limited.

**Table 1:** Nonspecific and tumor-targeted fluorescence agents used during TORS for HNC

Feature	Nonspecific Agent (ICG)	Tumor-Targeted Agents (e.g., Panitumumab-IRDye800CW)
Primary Utility	Perfusion, SLN mapping, and vascular permeability	Margin assessment and occult tumor detection
Specificity	Modest (71.9%) due to diffuse mucosal uptake and inflammation	High; leverages EGFR overexpression for tumor-specific contrast
Sensitivity	High (91.7%) but limited by low TBR in HNSCC	High; intraoperative TBR of ~ 10.7 in phase II cohorts
Firefly Mode	Standard Firefly (preserves grayscale anatomy)	Sensitive Firefly (suppresses background for low signals).
TBR	Average ~ 1.56	Mean intraoperative ~ 10.7
TORS Limitations	Failed to identify gross tumors or positive margins in some series	Lower absolute intensity; highly dependent on imaging geometry

## Clinical trials/studies relevant to TORS

A search of clinical-trials registries (U.S. and EU) revealed no active fluorescence-guided TORS trials. All fluorescence-guided HNC studies currently recruiting utilize open-field or endoscopic imaging rather than robotic platforms. Prospective studies and interim trial reports indicate that fluorescence can assist with intraoperative tumor delineation, facilitate SLN mapping/biopsy, and potentially reduce positive margins particularly for challenging deep or posterior planes. The reported performance varies with agent class, timing, imaging mode, and anatomic site. Current clinical evidence supporting fluorescence-guided TORS is still nascent, with available evidence primarily derived from early-phase, single-center studies and interim cohorts rather than definitive comparative trials. Accordingly, while our perspective is informed by direct institutional involvement, broader validation through multicenter trials is required before these workflows can be considered generalizable standards of care. *Interim synthesis*: In aggregate, nonspecific agents remain valuable for perfusion and lymphatic tasks, while targeted agents show the greatest promise for margin-focused, tumor-specific visualization in TORS. The literature is supportive but not yet definitive; device behavior and optical physics frequently determine success or failure at the console. This underscores the importance of the technical guidance and intraoperative strategies detailed in the subsequent discussion.

### Technical consideration and discussion

Effective integration of fluorescence imaging into TORS requires a thorough understanding of the optical principles, imaging-system behavior, and procedural variables that influence intraoperative interpretation. Unlike open surgery, robotic platforms constrain camera angle, working distance, and illumination geometry, making fluorescence behavior highly sensitive to technical factors. This section synthesizes the key considerations that govern fluorescence signal detection and interpretation during TORS, drawing from established optical principles, device specifications, published literature, and our intraoperative experience.

### Optical and tissue determinants of fluorescence signal

#### Tissue optics and exposure (what limits detection)

Both excitation and emission NIR photons are attenuated primarily due to absorption (mainly hemoglobin, water) and scattering in the tissue. Although the NIR window (700–900 nm) provides favorable tissue penetration and low autofluorescence compared with visible wavelengths, surgeons should recognize that fluorescence signal strength decreases exponentially with depth, and diminished emission may reflect physics rather than absence of tumor. This constraint is particularly relevant in the oropharynx and tongue base, where deep tumor margins often contribute to positive resections. Furthermore, spatial resolution drops with depth due to the ‘averaging effect’ and the ‘inverse square law’ reduction of the signal associated with longer working distance [23–25]. 

#### Relevance to TORS margin assessment

Deep or irregular margins common in tonsillar pillars, glosso-tonsillar sulcus, and lateral pharyngeal wall require careful interpretation. Faint or absent fluorescence does not necessarily indicate negative margins, especially near cauterized surfaces or highly vascular regions.

### Firefly imaging system and its integration with robotic platform: mode selection (Standard vs. Sensitive) and implications

The da Vinci Xi platform enables rapid toggling between two NIR imaging modes, *Standard Firefly* and *Sensitive Firefly*, which differ in illumination strategy, background suppression and fluorescence processing. Correct mode selection significantly impacts the detectability of tumor-targeted tracers. Standard Firefly mode blends onto a grayscale background, preserving anatomic context, and is well suited to nonspecific agents like ICG. Sensitive Firefly Mode introduces additional optical processing that increases the sensitivity to weak fluorescent signals typical of tumor-targeted tracers. This mode removes background visible illumination, reduces ‘white-light artifacts’ (reflection), and increases the fluorescence dynamic range. The Sensitive Firefly mode also provides access to the *Firefly Black Point* control, which sets the minimum threshold of displayed fluorescence as discussed further below. The trade-off is loss of tool control, darker field visualization, and reduced anatomic context. Key points differentiating the two modes are summarized in Supplemental Table 1.

Supplemental Fig. 1 shows the comparison of the two modes in ‘in-vivo & ex-vivo setting’. Meershoek et al. showed the spectral properties of the system’s light sources in white light mode (Standard Firefly) vs. fluorescence mode (Sensitive Firefly). They plot the blue-illumination peak (~ 460 nm) in white light mode and showed that in fluorescence mode the blue‐light intensity is reduced substantially while the NIR excitation peak (~ 800 nm, matches ICG fluorescence) is dominant [26]. Mastery of mode selection, along with awareness of field nonuniformity, gain presets, black-point thresholding, and autoscaling, is essential to avoid misinterpretation. The combination of targeted agents with Sensitive Firefly is particularly impactful for TORS, provided that workflow standardization (e.g., timing, scope geometry, cautery minimization before assessment) is enforced [23]. 

### Key practice

Surgeons should toggle between Standard (fluorescence + white light anatomy) and Sensitive Firefly (black background/sensitive fluorescence) modes in real-time to confirm fluorescence patterns while maintaining orientation. This improves the ability to see faint signals or differentiate signal from background. Knowledge and use of these modes helps the surgeon understand that any *residual visible light reflections or glints* seen in fluorescence mode may be *imaging artifacts* (e.g., from instrument reflection, specular tissue) rather than part of the intended illumination.

### Imaging geometry (exposure): scope angle, working distance, and camera motion

Consistency in fluorescence imaging and signal interpretation is strongly influenced by camera imaging geometry factors including angle and exposure, working distance to the tissue, and motion during the procedure. When tumors are located on the lateral pharyngeal wall, even minor adjustments in the robotic arm’s orientation can dramatically alter the field of view (FOV) and fluorescence intensity.

#### Scope angle and perpendicularity

Head & neck TORS procedures use 0- and 30-degree endoscopes as standard optics for imaging narrow oropharyngeal corridors or around curved anatomy (tongue base, supraglottic structures), respectively. Although the da Vinci Firefly system does not provide angle-corrected illumination, fluorescence imaging physics remains consistent with the broader NIR fluorescence literature, where the perpendicular orientation maximizes excitation efficiency and emission capture [20, 23, 27]. Van den Bos et al. provided an overview of the factors influencing the fluorescence intensity of fluorescence imaging with ICG, primarily focused on NIR fluorescence-guided cholangiography [27]. Among the concepts they discussed (fluorescence dye and concentration, timing of administration, penetration depth, distance to the target, angle between the laparoscope and the tissue, differences between imaging systems, and interpretation of the signal), they saw that some of the articles referred to using a 0-degree laparoscope, while others used a 30-degree laparoscope, which may be of influence on the angle of examination and the perceived fluorescence. An important note here is that da Vinci’s latest SP system, which is currently the most commonly used robot for TORS, does not require the use of zero- or 30-degree endoscopes due to the camera’s ‘cobra function’ with two joints that enables maneuvering the camera to the desired angle.

#### Working distance

Da Vinci Xi uses “chip-on-tip” system where the image sensor sits at the distal tip of the endoscope, instead of farther back in the scope body. This provides a wide-angle optics arrangement that minimizes the working distance to the surgical field and improves excitation and photon capture for fluorescence imaging [26]. In our experience with this endoscope system, optimal working distance is at ~ 5 cm. Increased distance decreases signal via inverse-square law and scattering effects, creating the illusion of weaker fluorescence [27]. Supplemental Fig. 2 shows fluorescence intensity variations with change in scope angle and working distance. Maintaining a consistent camera geometry during margin interrogation is recommended.

#### Camera movement and autoscaling (auto-contrast, auto-gain mapping)

**Fig. 1:**
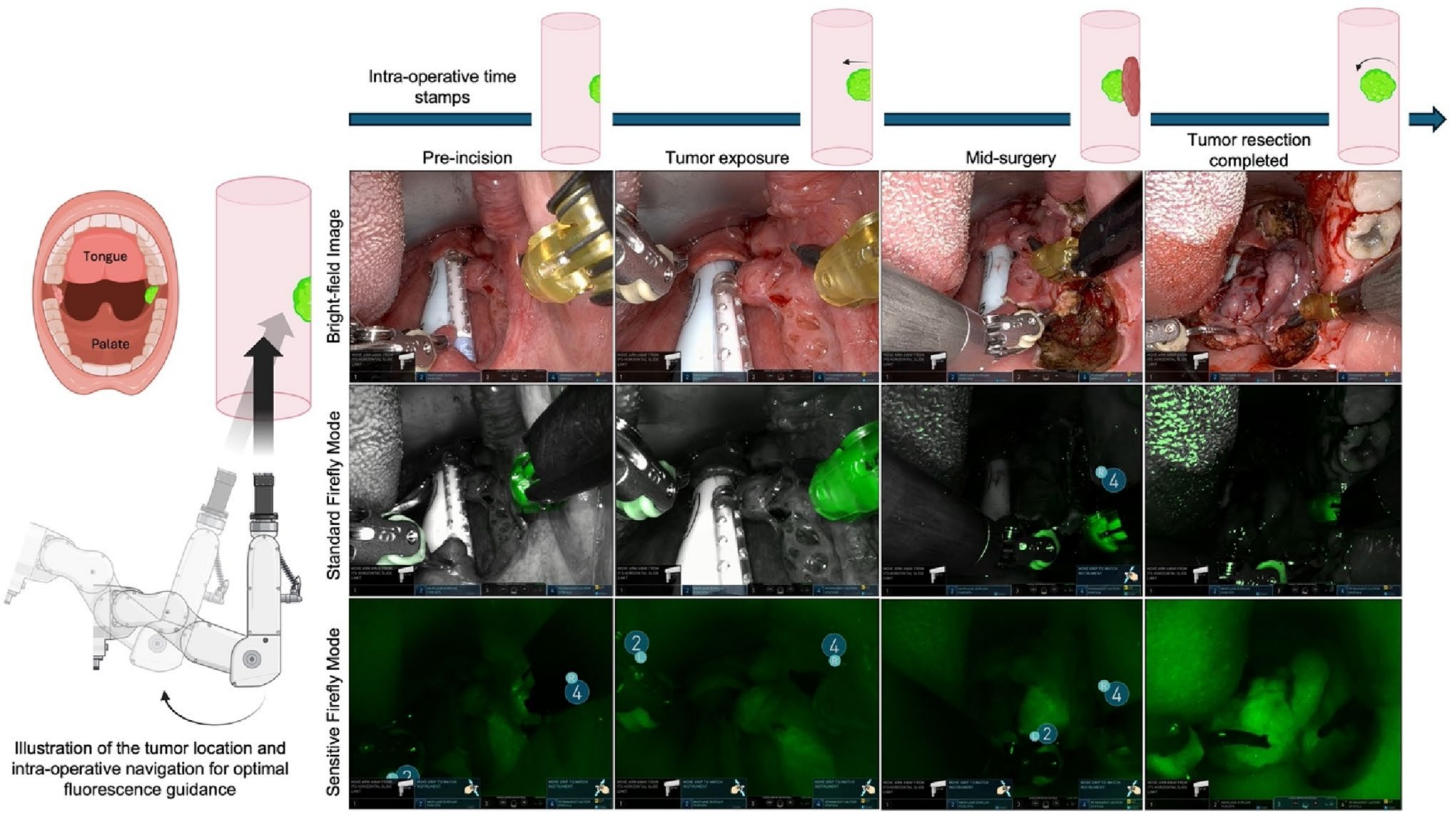
Illustration of tumor location and trans-oral imaging compared to imaging through a cylindrical tube and the need for manipulation of robotic arms for optimal imaging conditions shown on the left side. Intraoperative images on the right side show each column at critical time-points during the surgery and the corresponding Standard and Sensitive Firefly Mode fluorescence Images. The pre-incision intraoperative images show moderate fluorescence contrast in the tonsillar tumor due to overlying mucosa. As surgery progresses to expose the tumor, the robotic endoscope is advanced closer to the surgical plane and tilted perpendicularly to the tumor surface (as illustrated on the left), thereby increasing fluorescence contrast

Autoscaling dynamically adjusts brightness based on the brightest and dimmest pixel intensities in the FOV to maximize visual contrast. If the raw intensity values are small, the display will ‘stretch’ them to use the full dynamic range of the software. Tumor fluorescence signals in surgery vary by orders of magnitude from weak/faint to bright hotspots. As the camera moves between high- and low-signal regions, the system may artificially brighten or dim the scene, mimicking biological changes. This can create misleading impressions of increasing or decreasing fluorescence intensity. Camera movements, exposure, and imaging geometry leading to optimal fluorescence guidance are illustrated in Fig. 1.

### Imaging system controls: gain, black-point thresholding, and field uniformity

#### Gain presets

Gain is the digital or analog amplification factor applied to the camera sensor’s signal. Increasing the gain doesn’t create new light but simply amplifies the detected photon signal across all image pixels so faint fluorescence can be visualized. The presets are predefined levels to adjust gain (low, medium, or high) in real time. Each preset optimizes the exposure time, camera gain, and display contrast to balance brightness and noise.

- **High gain**: Enhances weak signals but increases noise.
- **Low gain**: Prevents overexposure, preserves bright signals, but risks missing subtle fluorescence.

Surgeons can quickly switch the gain presets to adapt to variable fluorescence intensities and ambient lighting conditions in the operating rooms to see both strong and faint signals without saturating the image or losing details. Presets also help to enhance reproducibility by keeping the intensity values comparable across operators, patients and imaging sessions. It is important to note gain levels when interpreting or recording images, as amplification may be misinterpreted as biological brightness in post-processing of these recordings.

#### Black-point imaging

A thresholding feature only active on the Sensitive Firefly mode suppresses the background and renders only the fluorescence signal visible, while remaining pixels are assigned negligible values. ‘Black point’ thresholding, which the operator can adjust through an intensity slider, defines the minimum intensity level that will be shown as pure black with fluorescence mapped to bright tones (gray/green/pseudo color). This function is useful for detecting faint tumor signals but can exaggerate noise, obscure anatomical context, and worsen peripheral falloff effects. Since this mode is also affected by the illumination fallout discussed in the following paragraph, peripheral field non-uniformity becomes more visible, making margin assessment unreliable. *Relevance to TORS*: Surgeons must avoid over-reliance on this function for margin decisions.

#### Field uniformity (illumination uniformity/flat-field response)

Field uniformity is the measure of how evenly the excitation light and detector sensitivity are distributed across the FOV of an imaging system. The illumination fibers or LEDs in most endoscopic systems non-uniformly distribute stronger excitation in the center than at the edges, resulting in non-uniform signal brightening at the center (potential overestimation of fluorescence) and signal falloff at the edges of the FOV (potential underestimation of fluorescence). Spectral shifts, filter and detector efficiency, drop of signal due to angled filters, tissue curvature, and angle of the camera can contribute to non-uniformity that may influence interpretation of perfusion or tumor fluorescence. Meershoek et al. reported that Firefly systems on both the da Vinci Si and Xi platforms exhibit distinct differences in excitation efficiency and detector sensitivity across the field, with peripheral regions showing reduced fluorescent output [26], while Gioux et al. similarly describe how optical design, including filter angle sensitivity, spectral shifts, and working distance-dependent changes, contributes to non-uniform signal distribution [28]. A recent study using phantoms for NIR fluorescence uniformity and distortion assessment demonstrated that fluorescence imaging systems exhibit substantial variation in field uniformity, with some commercial devices maintaining ≥ 60% of maximum intensity across most of the field, while others show less than half of the field meeting this threshold. While flat-field correction can make images appear more uniform, it may distort quantitative measurements, emphasizing the need to characterize and rely on the most uniform regions of the FOV rather than correcting non uniformity post-hoc [29]. This contributes to apparent brightness biases in assessments, complicates thresholding and heatmap generations (post-processing) and reduces margin detection sensitivity. Flat-field correction on ex-vivo specimens provides a practical method to normalize spatial variation and improve quantitative margin evaluation post-resection.

#### Practical recommendation

Evaluate fluorescence at the center of the field, where uniformity and detector response are most consistent.

### Biological and procedural artifacts affecting interpretation

Artifacts and interpretation challenges arise from both *optical and biological factors. Specular reflections* from moist mucosa or metallic instruments can mimic fluorescence. *Heterogeneous tissue perfusion*, blood pooling, or incomplete dye delivery can also confound interpretation, creating patchy or absent fluorescence that does not necessarily indicate lack of tumor or perfusion. *Depth attenuation* is another key limitation, as NIR fluorescence penetrates only a few millimeters, so deep-seated tumor deposits may remain invisible.

#### Autofluorescence

Native autofluorescence from collagen, cauterized tissue, or inflamed mucosa may create low-level false positives. This is particularly common in previously treated fields or at the base of tongue. Autofluorescence and blood can also reduce the contrast, making highly vascular or blood-stained fields mask the signal.

#### Cautery-related changes

**Fig. 2:**
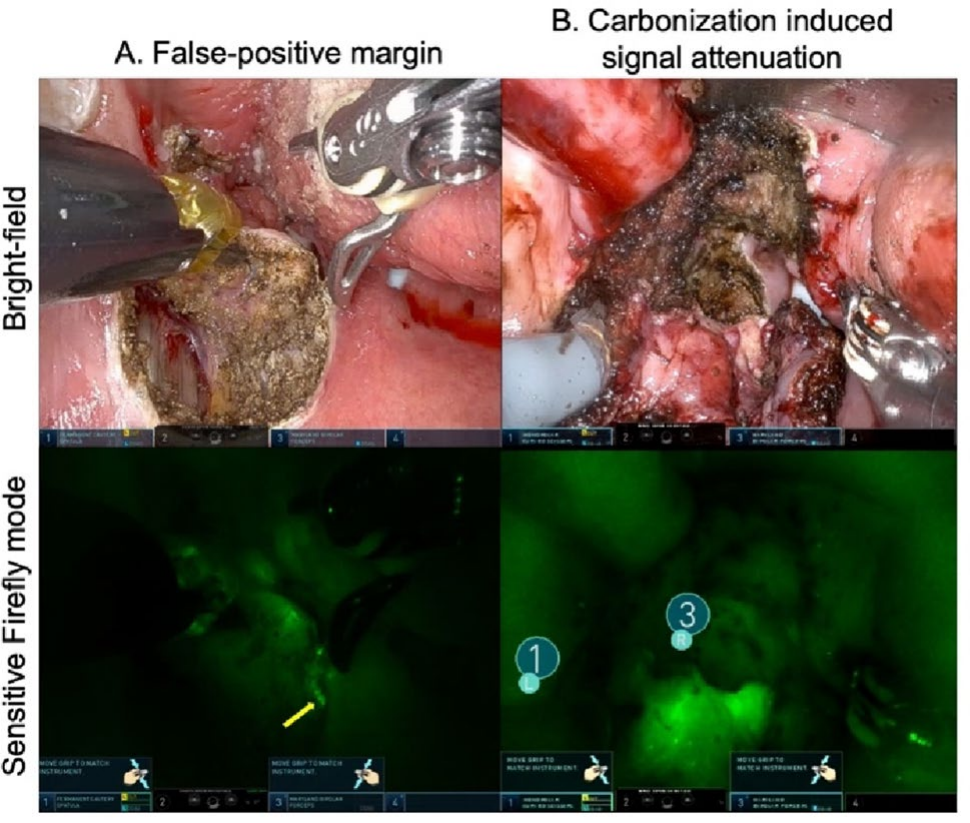
Cautery-induced tissue changes. (**A**) shows image of initial stage of surgery, corresponding fluorescence image shows the yellow arrow pointing at the fluorescence related to false-positive margins due to optical, biological and tissue autofluorescence factors. (**B**) shows eschar related to thermal tissue injury obscuring the tumor signal

Thermal injury alters scattering, quenches intrinsic fluorescence, and carbonizes tissue. Cauterized surfaces often appear falsely negative, particularly at resection margins. Conversely, retained eschar or reflective char can produce focal “hot spots.” This creates a significant challenge during intraoperative margin evaluation. Residual thermal coagulum may trap fluorophore or alter its emission, causing faint fluorescence in non-tumorous zones. Thus, interpretation of fluorescence near cauterized tissue must be cautious regarding signal loss that may reflect thermal-injury related changes, not true clearance. Fluorescence changes associated with cautery-induced tissue injury are illustrated in Fig. 2.

Summary of recommendations to account for biological artifacts during fluorescence assessment:

a) Use cold instruments for the final millimeters of margin dissection.

b) Irrigate or wipe before assessing fluorescence.

c) Correlate fluorescence with white-light visualization.

d) Integrating fluorescence findings with anatomical knowledge helps preserve the diagnostic value of fluorescence guidance in real time; when feasible, frozen-section pathology should be used as an additional safeguard against misinterpretation.

### In-field reference strategy

**Fig. 3:**
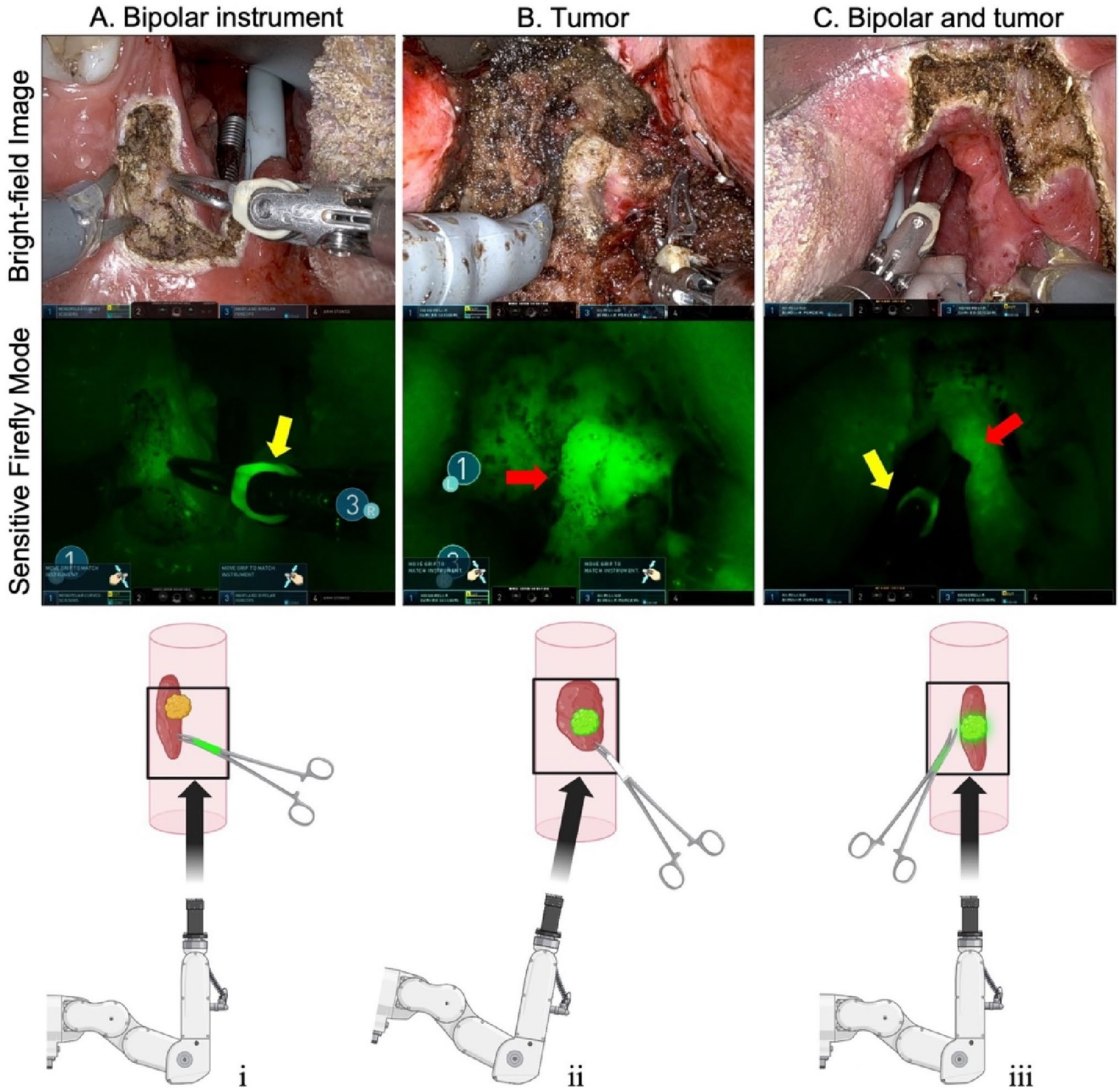
*In-field reference* - Autofluorescence of the bipolar cautery of the robotic arm used as in-field reference during intraoperative navigation based on the object in the center of the FOV. (**A**) shows bipolar instrument alone in the FOV, scaled to the brightest pixel (yellow arrow). (B) shows tumor alone, scaled to the brightest pixels (red arrow) and the (**C**) shows bipolar instrument (yellow arrow) and tumor (red arrow), brightest object (tumor - red arrow) scaled to the brightest pixels. Illustrations (i, ii, iii) show the FOV (represented by the black rectangle) with bipolar instrument (i), tumor (ii), and tumor and bipolar instrument (iii) as regions of interest focused on the perpendicular line angle of the camera

Robotic instruments, particularly the da Vinci Xi bipolar forceps, can exhibit reproducible autofluorescence that can be leveraged as an ‘in-field reference’ standard to contextualize tissue signal intensity during fluorescence-guided TORS. Stone et al. [22] observed that instrument autofluorescence varies with the system’s autoscaling behavior, yet remains sufficiently stable to support relative normalization of tissue fluorescence. In our workflow, this concept is illustrated in Fig. 3, which demonstrates how instrument-anchored normalization stabilizes intraoperative interpretation across changing fields of view. When autofluorescence of the bipolar tool is alone in the FOV (Fig. 3A, yellow arrow), the autoscaling makes the bipolar pixels the brightest (highest dynamic range values). However, when the tumor is alone in the FOV, the autoscaling makes it the brightest (Fig. 3B, red arrow). When both tumor and bipolar are in the FOV, the pixels with the highest absolute intensity are scaled the brightest. When this is the tumor, the bipolar is scaled lower (Fig. 3C, red and yellow arrows). Stone et al. achieved strong diagnostic discrimination between OPSCC and normal tissue (AUC 0.975, 90.9% sensitivity, 96.0% specificity) by normalizing tissue mean fluorescence intensity to instrument autofluorescence (tissue-to-instrument standard fluorescence ratio, SFR) in intraoperative robot fluorescence views. They demonstrated that when a tissue of suspicion in the FOV exhibited > 1.37 SFR over the bipolar signal, the suspicious tissue was cancer 96% of the time [22]. Collectively, these findings suggest that an internal instrument fluorescence reference can reduce interpretive variability introduced by autoscaling and improve confidence when evaluating low-intensity signals at tumor borders and margins, particularly with tumor-targeted tracers.

### Integration of tumor-targeted agents in TORS

Targeted tracers (panitumumab-IRDye800CW, cetuximab-IRDye800CW) offer superior tumor contrast but also present unique challenges, including lower overall signal intensity compared with ICG, time-dependent biodistribution and clearance, the need for Sensitive Firefly mode for adequate visualization, and greater susceptibility to illumination conditions and autoscaling artifacts. Despite these limitations, these agents hold strong promise for enhancing oncologic precision, provided that standardized imaging protocols, optimized timing strategies, and structured intraoperative workflows are implemented.

### Inter-patient variability and pharmacokinetic considerations

In addition to optical and technical factors, fluorescence signal characteristics are influenced by inter-patient biological variability and tracer pharmacokinetics. For both nonspecific (ICG) and tumor-targeted agents (e.g., panitumumab-IRDye800CW), factors such as tumor biology, receptor expression heterogeneity, vascular permeability, and systemic clearance can affect signal intensity, TBR, and diagnostic performance between patients [16, 30]. Importantly, non-malignant conditions including dysplasia, inflammation, and reactive tissue changes may alter tracer uptake or autofluorescence, potentially confounding interpretation and reducing specificity [31, 32]. While demographic variables such as age, sex, and ethnicity may indirectly influence these parameters through differences in tissue composition and pharmacokinetics, current clinical studies have not demonstrated consistent or clinically actionable effects on fluorescence contrast [33]. 

Timing of image acquisition relative to tracer administration further impacts contrast. Optimal imaging windows typically occur 1–4 days after systemic administration of antibody-based imaging agents [33], while ICG fluorescence imaging occurs immediately after infusion during vascular imaging or 1–2 days after systemic administration for delayed tumor visualization (“second window ICG imaging”) [9]. These biological and pharmacologic variables have been explored in dedicated translational and clinical studies and underscore the need for standardized dosing and imaging protocols when applying fluorescence guidance in TORS, including prospective evaluations with tumor-targeted agents [22]. 

## Overall summary

Fluorescence-guided TORS provides a valuable visual adjunct that compensates for limitations in tactile feedback and depth perception inherent to robotic surgery. However, accurate interpretation requires familiarity with optical physics, device behavior, and procedural nuances. When these factors are understood and incorporated into surgical workflow, fluorescence imaging has the potential to improve margin assessment, enhance tumor localization, and support more precise oncologic surgery. Table 2 gives a summary of observed fluorescence perturbations and suggested mitigation strategies.

**Table 2:** Overview of technical aspects for clinical consideration:

Factor	Mechanism	Surgical strategies
Camera angle and exposure	Affects excitation/emission capture	Keep FOV as perpendicular as feasible
Working distance	Inverse-square fallout, scattering	Optimize to ~ 5 cm, avoid drifting away from target
Field uniformity	Central brightening, edge falloff	Judge margins at the image center
Gain presets	Amplifies both signal and noise	Note gain, avoid overinterpreting brightness
Black-point (threshold)	Hides background; boosts contrast	Use sparingly, reorient with grayscale anatomy when in doubt
Autoscaling (auto-contrast)	Dynamic display rescaling	Be aware of false changes with camera motion
In-field reference	Tool autofluorescence as anchor	Normalize relative intensity intraoperatively
Artifacts (specular, cautery, autofluorescence)	Quenching, char reflections	Minimize cautery OR avoid overinterpreting fluorescence in cauterized regions, irrigate before imaging

## Limitations

This is a narrative review and does not apply systematic screening, bias assessment, or meta-analytic methods, and therefore, publication bias and selective reporting in the source literature may influence our synthesis. Clinical studies of fluorescence during TORS in HNC remain heterogeneous with respect to patient selection, tracer class/dose, infusion-to-surgery interval, imaging mode, and outcome definitions, limiting cross-study comparability. Data specific to robotic workflows are still emerging, and many reports do not detail critical imaging parameters that materially affect signal interpretation. Finally, we incorporate institutional experiential observations to highlight practical nuances; while clinically informative, these insights are anecdotal and require validation in prospective, standardized studies.

## Future directions

Current trends in the medical, scientific, and industrial communities indicate continued expansion of applications and development of fluorescence imaging during robotic surgery. The following summarizes key developments and considerations for widespread implementation of fluorescence imaging during TORS.

### Considerations for single-port (SP) robotic platforms

While much of the published technical characterization of fluorescence imaging in head and neck robotic surgery has been performed using the da Vinci Xi platform, contemporary TORS practice is increasingly transitioning toward the single-port (SP) system. The SP platform offers important ergonomic advantages in the confined anatomy of the upper aerodigestive tract, including improved access through a single cannula, flexible “cobra” camera articulation, and enhanced visualization of curved or recessed subsites such as the tongue base and supraglottis [13]. These features can facilitate more consistent exposure and camera positioning, which are critical determinants of fluorescence signal quality. The FDA recently approved the da Vinci SP Firefly Imaging System, which now incorporates both Standard Firefly and Sensitive Firefly imaging modes [34]. This development brings SP fluorescence functionality closer to that previously described for Xi-based systems and may improve visualization of tumor-targeted fluorophores during TORS, though the optical principles outlined throughout this review remain applicable across robotic platforms.

### Fluorescence agent development and access

Next-generation targeted tracers (including dual-target or activatable probes) may enhance specificity and reduce background. Streamlined regulatory pathways and multicenter trials tailored to robotic workflows are needed to expand clinical availability.

Future development in fluorescence-guided TORS is likely to involve not only improved exogenous tracers but also integration of complementary endogenous and fluorescence lifetime-based optical signals. There is growing interest in leveraging endogenous tissue fluorophores and their autofluorescence potential which include collagen, nicotinamide adenine dinucleotide (NAD(P)H), flavin adenine dinucleotide (FAD), and porphyrins for intraoperative optical guidance. These intrinsic fluorophores enable label-free assessment of tissue metabolic state, structural composition, and biochemical changes associated with malignant transformation which is also referred as photodynamic diagnosis by several authors [35–38]. In particular, fluorescence lifetime imaging (FLIm) provides contrast based on fluorophore decay kinetics rather than intensity alone, offering resilience to variations in illumination, tissue geometry, and signal attenuation that commonly affect intensity-based imaging in robotic platforms. Early implementations of fiber-based FLIm systems integrated with robotic instruments suggest feasibility for real-time discrimination between tumor and normal tissues in confined surgical fields [35, 38–40]. 

For TORS, these approaches are particularly attractive given the sensitivity of intensity-based fluorescence to imaging geometry (e.g., working distance, angle, and autoscaling), as discussed in this review. Endogenous and exogenous fluorescence techniques may be complementary: targeted tracers can provide high-specificity tumor localization, while endogenous or lifetime-based signals may offer orthogonal information related to tissue metabolism and microenvironment. The integration of these modalities potentially through multispectral or hybrid imaging platforms represents a promising direction for improving margin assessment and reducing reliance on any single contrast mechanism [41, 42]. However, challenges remain, including signal interpretation complexity, limited penetration depth, hardware integration constraints, and the need for standardized acquisition and validation in robotic workflows.

### Standardized imaging protocols/terminologies

The field will benefit from consensus on FGS terminologies, tracer dosing/timing, required use of Sensitive Firefly for targeted agents, and minimum reporting standards. Embedding these parameters in operative notes and publications will improve reproducibility and enable pooled analyses.

### Quantification and analytics

Real-time quantification (e.g., relative fluorescence units normalized to an in-field reference), automated flat-field correction, and angle-aware calibration could stabilize interpretation across cases. Integration of on-board analytics (e.g., margin heatmaps robust to autoscaling) directly within the robotic console is a logical next step.

### Multimodal imaging

Combining fluorescence with augmented reality overlays, preoperative imaging (CT/MRI) co-registration, or complementary optical methods (e.g., multispectral) may improve subsurface margin assessment and anatomic orientation in confined corridors. Recent work demonstrates the feasibility of reconstructing 3D models of Firefly fluorescence from da Vinci stereo endoscope and registering and overlaying these models onto segmented pre-operative CT within an image guided display, achieving ~ 2–3 mm localization accuracy in a phantom model. This approach suggests a path toward augmented reality-style fluorescence navigation, including persistent display of short-lived ICG information and potential use of fluorescence features to assist robot–image registration [43]. A technical evaluation of Firefly cameras on the da Vinci S and Xi/X systems showed that clinically available hardware can support dual-dye (fluorescein + ICG) multi-wavelength imaging, and that the Xi/X platform provides superior excitation and detection sensitivity. These findings motivate future robotic workflows that incorporate multispectral fluorescence for simultaneous visualization of complementary structures (e.g., lymphatics and vasculature/tumor) [26]. 

As fluorescence imaging becomes increasingly integrated into robotic workflows in the clinic, we also propose standardization of training and imaging workflows during TORS that maintain rigor, reproducibility, and patient-centered outcomes. Surgeons typically train in TORS through a staged pathway that includes didactic instruction, dry-lab simulation, cadaveric dissection, and proctored clinical cases, often following structured curricula developed at high-volume centers. Adopting similar training criteria is required for fluorescence-specific TORS competencies. Device manuals emphasize that operator-set modes and geometry materially change the perceived signal. As recommended by the scientific community, wide-field optical imaging requires calibration literacy and standardized acquisition techniques for reliable interpretation.

### Certification & advanced training

Adopting fluorescence-guided TORS should incorporate formal certification modules that cover the physics of NIR fluorescence imaging, system specific behaviors, performance limits, and calibration concepts. Programs can use low-risk bench and video simulations and phantom-assisted exercises. An institutional credentialing framework can help build such training programs.

Recommended documentation standards (minimum dataset):

• Checklist for fluorescence guided TORS (similar to surgical checklists) - Agent, dosage timing, platform setting (mode, gain, background setting).

• Standardized pre-assessment before any fluorescence readout is recorded or acted on. This can help localize the area of interest and find satellite tumor deposits.

• 2-person confirmation whether the ROI is centered, view is perpendicular, and the working length is optimal. Future iterations of robotic platforms may have a time-of-flight function, which is a laser assisted measure of the working length that can aid standardization of the focal distance between the camera and the tissue. Currently, manually adjusting the working length for optimal fluorescence is needed at every major time-point during the surgery.

• At each fluorescence readout: Tumor screening, margin check, cavity survey images are captured and any factors that could bias intensity (recent cautery, blood pooling) are documented along with use of in-field fluorescence references.

The fluorescence image seen is a function of illumination + optics + processing, not just biology. Standardizing fluorescence acquisition variables, including timing during the surgery, instrument settings, and detector geometry relative to the surgical field, is central to making fluorescence interpretation reliable across operators and centers. This remains the pre-requisite for effective intra-operative guidance and multicenter trials.

## Conclusion

Fluorescence-guided robotic surgery represents a significant advancement in the intraoperative management of HNC. By addressing the limitations of robotic platforms, particularly the absence of tactile feedback and direct visualization, fluorescence imaging enhances the surgeon’s ability to identify tumor margins, assess resection completeness, and perform sentinel lymph node mapping with greater precision. The integration of tumor-targeted agents such as panitumumab-IRDye800CW, along with optimized imaging platforms like the Sensitive Firefly system, has enabled real-time visualization of tumor tissue during TORS. In vivo and ex vivo imaging workflows, when standardized, offer complementary insights that can improve intraoperative decision-making and potentially reduce positive margin rates.

Despite promising early-phase trials and technical innovations, several challenges remain. These include variability in imaging protocols, limited data specific to robotic applications, and the need for regulatory pathways to support broader clinical adoption. Furthermore, the interpretation of fluorescence signals requires careful consideration of imaging physics, scope settings, and potential artifacts such as cautery effects or autoscaling biases. Looking ahead, the field is poised for rapid evolution. Future directions include the development of multimodal imaging agents, AI-assisted fluorescence quantification, integration of real-time analytics into robotic platforms, and multi-spectral imaging. As evidence accumulates and workflows mature, fluorescence-guided robotic surgery has the potential to become a standard adjunct in oncologic surgery, improving both oncologic and functional outcomes for patients with HNC.

Practical Synthesis: Pearls for Intraoperative Fluorescence-Guided TORS: (Supplemental Figs. 2 & 3)

These technical factors collectively shape the reliability of signal interpretation during robotic surgery.

1. Position the scope as perpendicular as possible to the tissue at the ideal working length. (Supplemental Fig. 2)

2. Center the region of interest in the field to avoid edge falloff.

3. Interpret fluorescence on both Standard and Sensitive modes.

(In the Supplemental Fig. 3, the difference between upper and lower panels and illustrations shows the above points in real time clinical practice).

4. Use instrument autofluorescence as an internal in-field reference (Supplemental Fig. 3C).

5. Minimize cautery before fluorescence assessment. (Supplemental Figs. 2 and 3)

6. Recognize autoscaling-induced brightness shifts during camera motion.

7. Document gain levels to contextualize signal intensity.

## Supplementary Information

Below is the link to the electronic supplementary material.

Supplementary Material 1

Supplementary Material 2

## Data Availability

The data that support the findings of this study are available on request from the corresponding author.
