# Supplementary material for: Considerations for transoral robotic surgery with fluorescence imaging: a narrative review
Source: J Robot Surg. 2026 Jul 27;20(1):754. doi: 10.1007/s11701-026-03724-8 (PMC13402249; doi:10.1007/s11701-026-03724-8)
Supplement: Supplementary file 1 — Supplementary Material 1 [file 11701_2026_3724_MOESM1_ESM.pptx]

## Slide 1
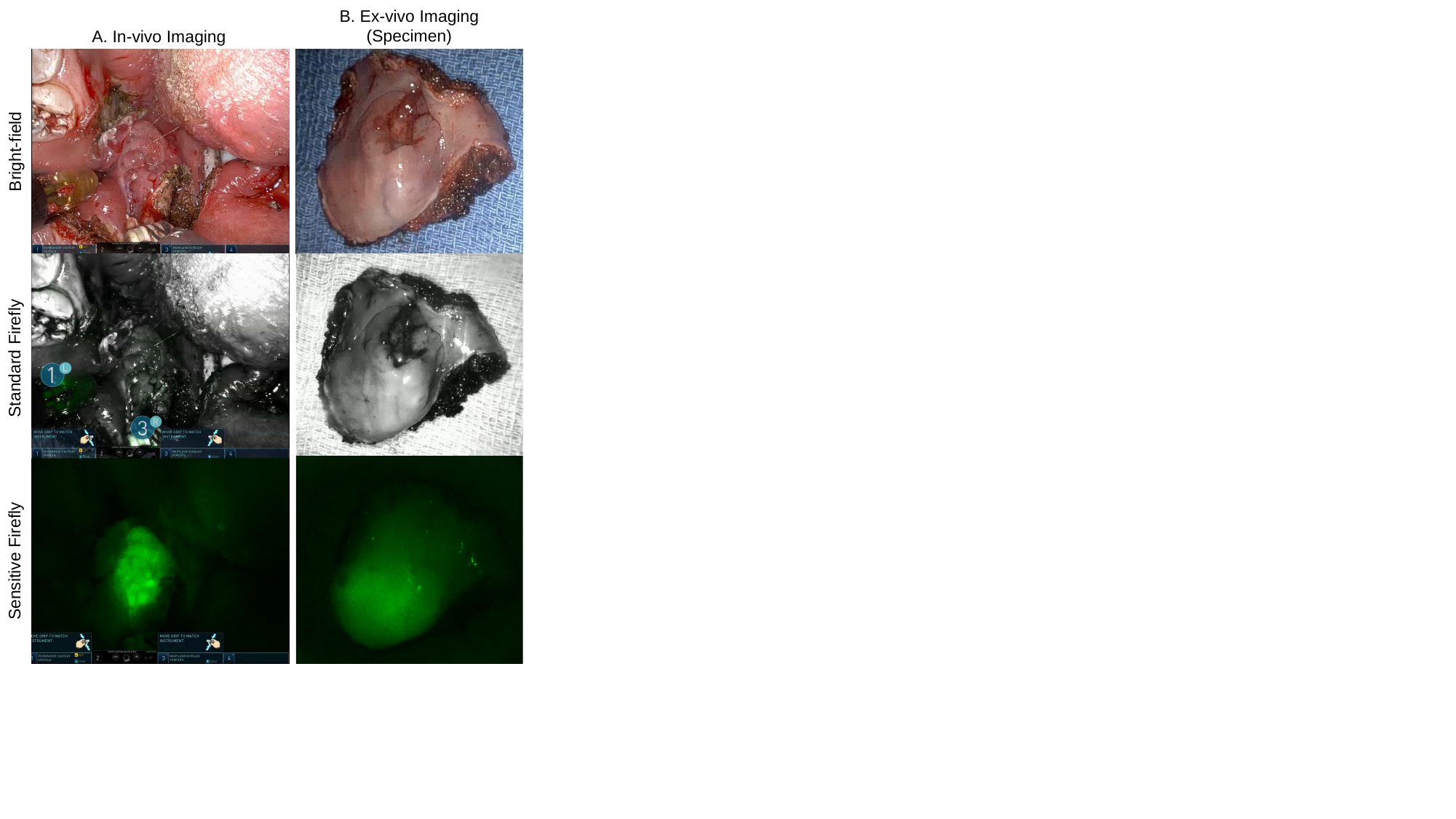

B. Ex-vivo Imaging
(Specimen)
A. In-vivo Imaging
Bright-field
Standard Firefly
Sensitive Firefly

## Slide 2
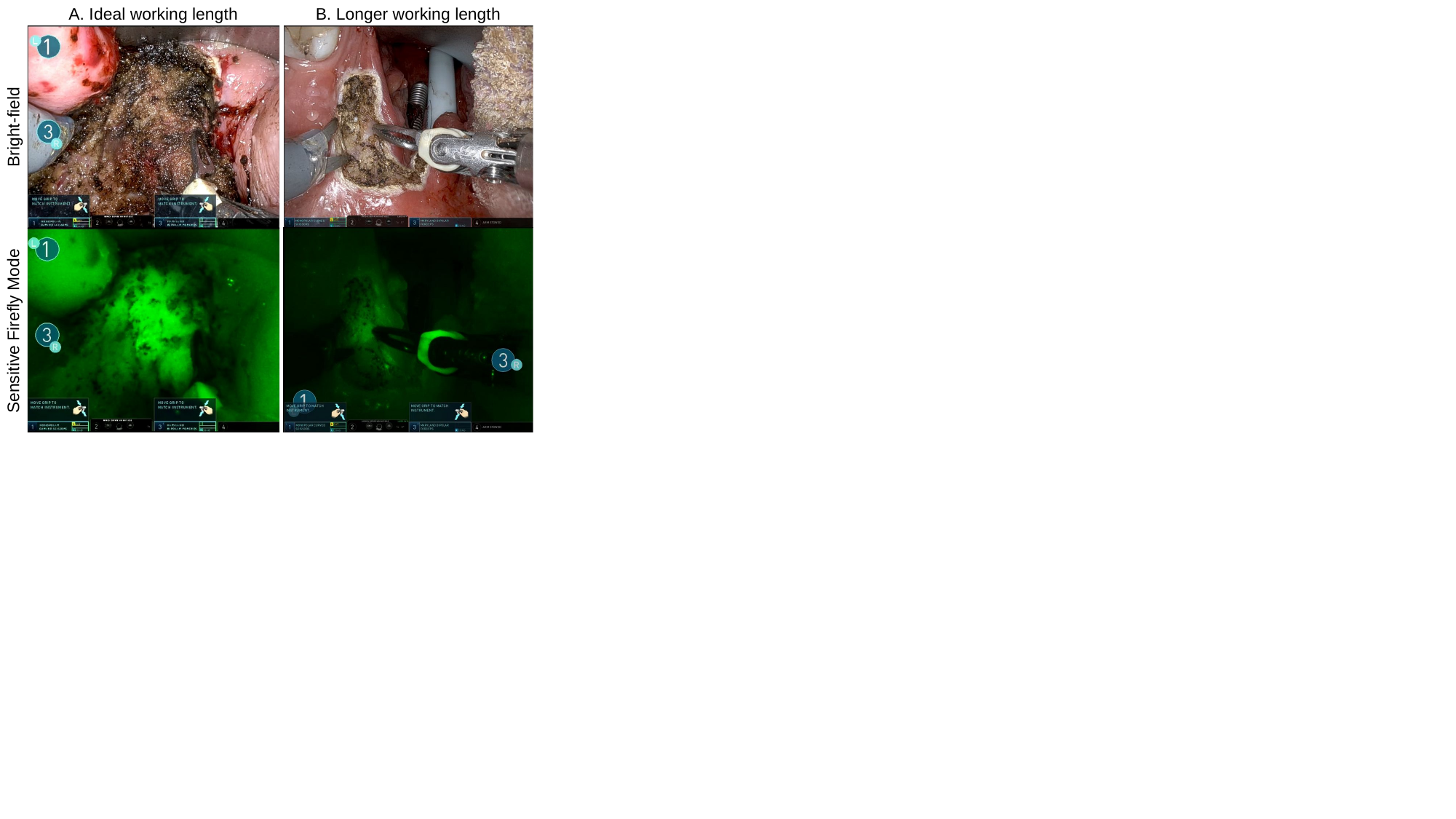

A. Ideal working length
B. Longer working length
Bright-field
Sensitive Firefly Mode

## Slide 3
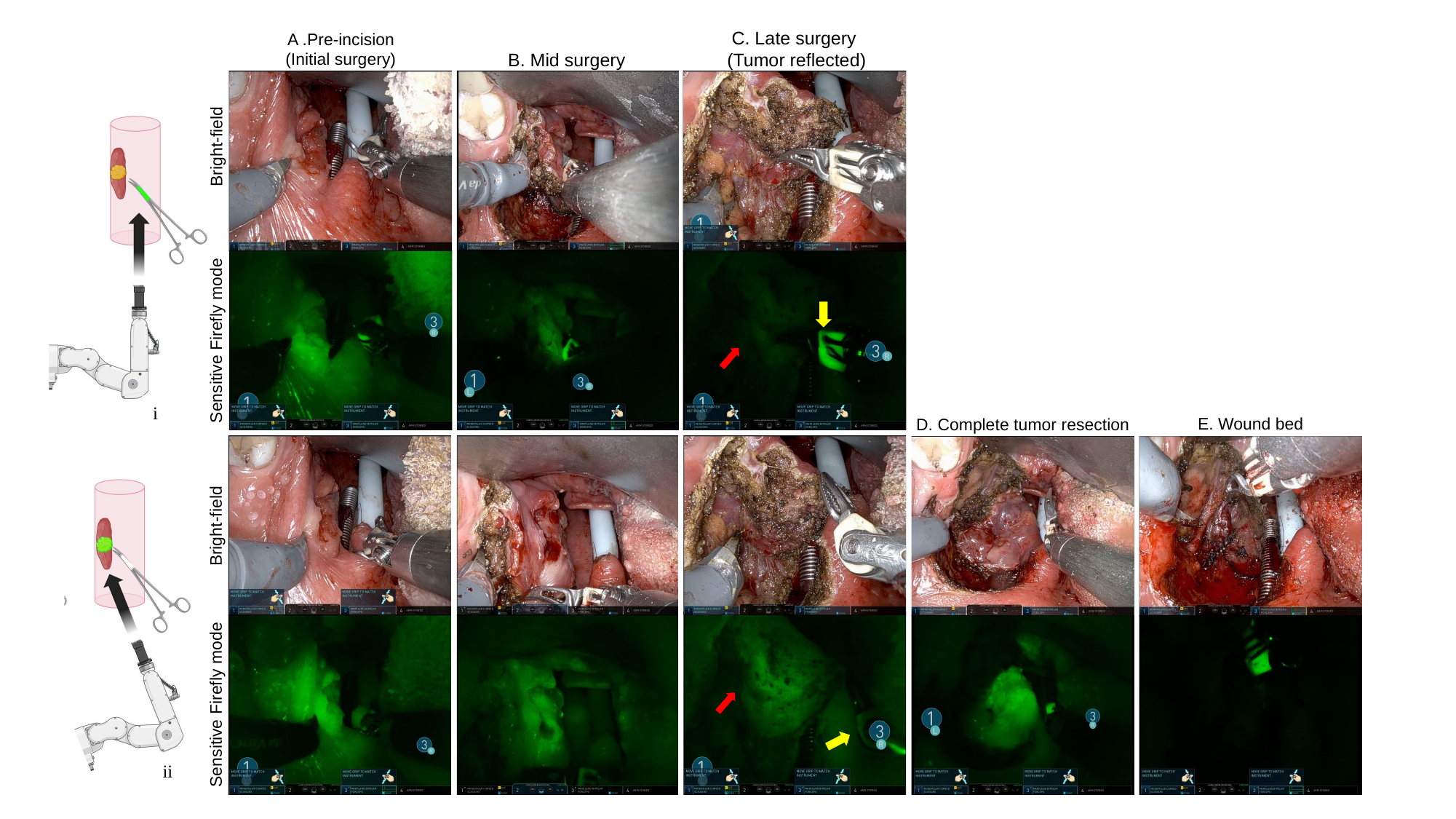

A .Pre-incision (Initial surgery)
C. Late surgery
(Tumor reflected)
B. Mid surgery
Bright-field
Sensitive Firefly mode
i
E. Wound bed
D. Complete tumor resection
Bright-field
Sensitive Firefly mode
ii
