# Supplementary material for: Considerations for transoral robotic surgery with fluorescence imaging: a narrative review
Source: J Robot Surg. 2026 Jul 27;20(1):754. doi: 10.1007/s11701-026-03724-8 (PMC13402249; doi:10.1007/s11701-026-03724-8)
Supplement: Supplementary file 2 — Supplementary Material 2 [file 11701_2026_3724_MOESM2_ESM.docx]

**
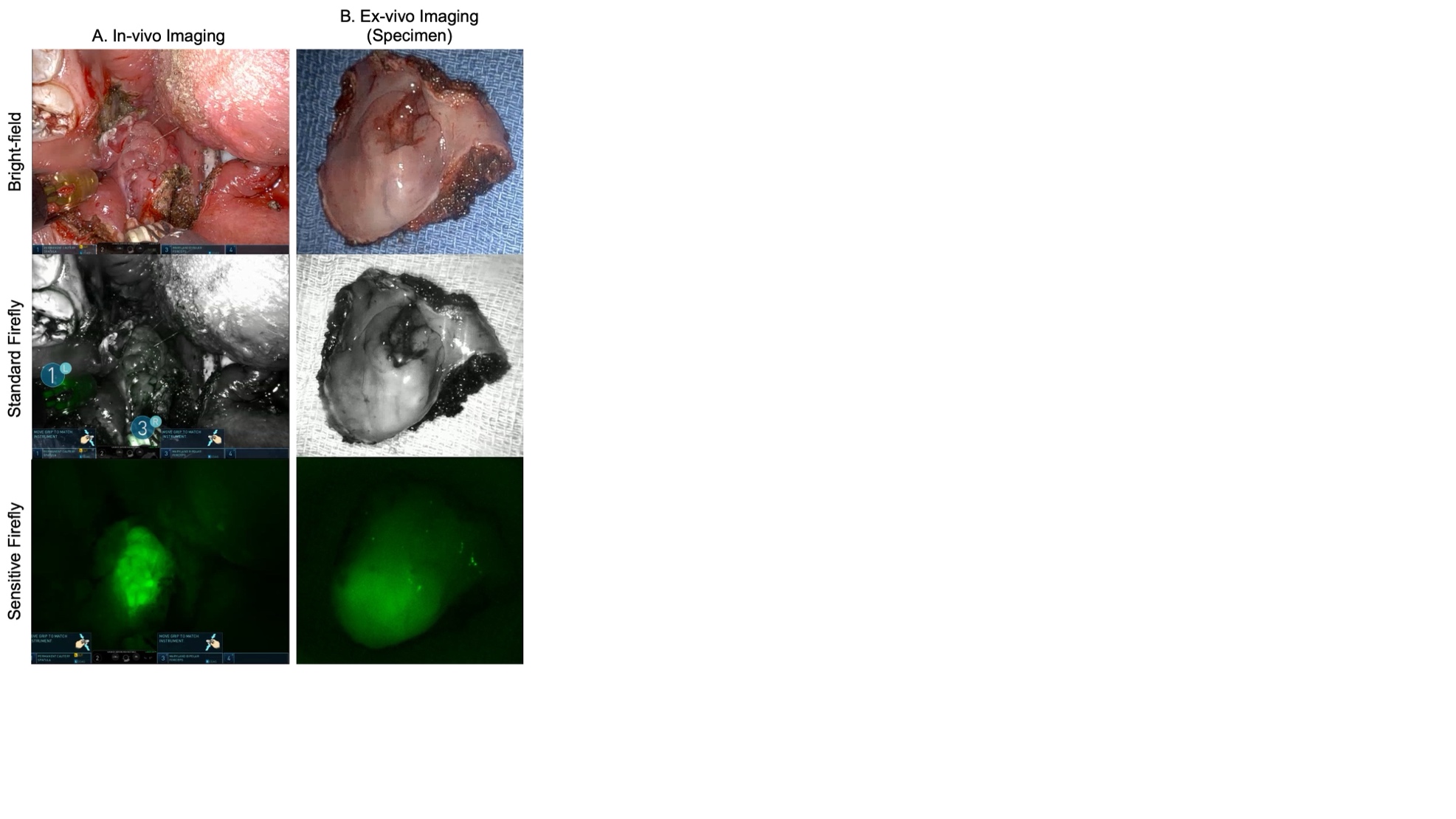
**

**Supplemental Figure 1: A (In-vivo) and B (Ex-vivo) shows the difference in fluorescence images in standard and sensitive firefly mode.** Specimen was imaged on the back-table using the da Vinci Xi endoscope camera. The camera was detached from the robotic arms and moved to the back-table to image the specimen and show the difference between the standard and the sensitive firefly mode in ex-vivo.


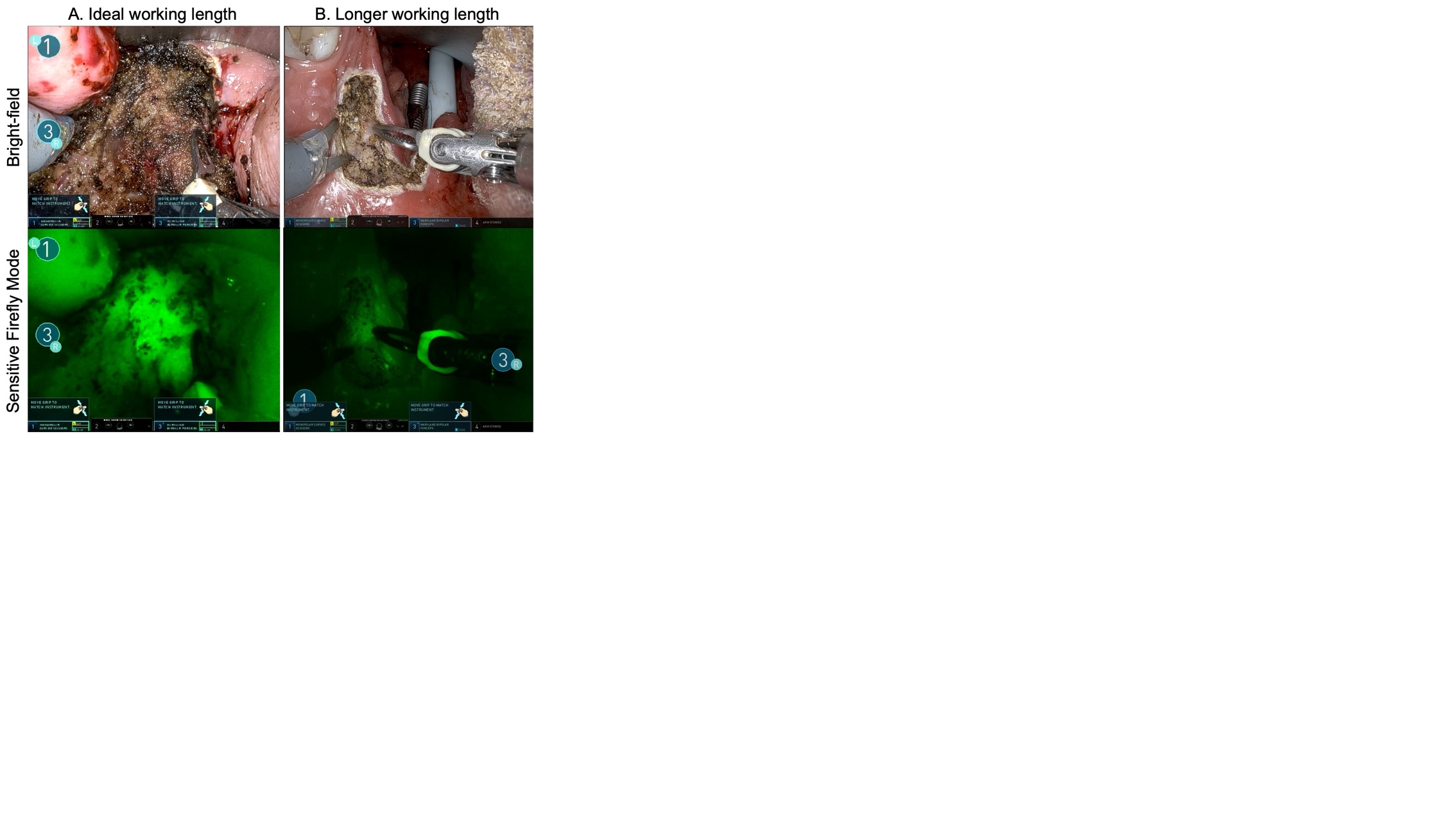


**Supplemental Figure 2**: Fluorescence imaging with the same during the initial dissection of the tumor with slightly different FOV (change in camera angulation) and working length (decreased). (A) shows images from a closer working length (~5 cm) and higher fluorescence intensity with exaggerated cautery induced artifacts. (B) shows images from a longer working distance and the bipolar instrument auto scaled to the brightest pixels. Note that same images in panel B appear figure 3 to illustrate the concept of in-field reference. We chose to use the same images to show the dynamic nature of these concepts. All the parameters discussed in the review are to be looked at simultaneously.


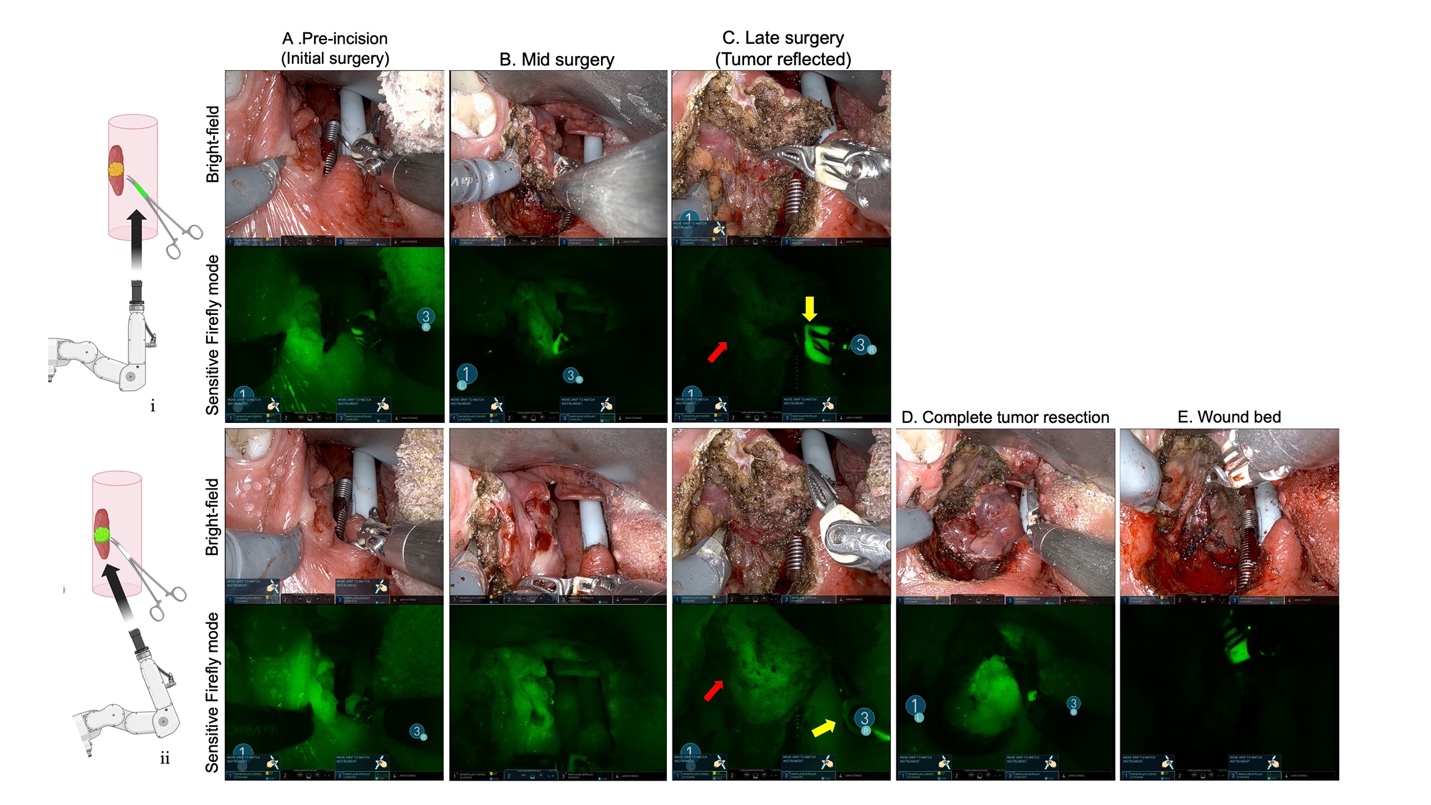


**Supplemental Figure 3**: **Complete case fluorescence guided robotic surgery workflow:** Pre-incision (tumor exposure by retraction of tissues using the monopolar cautery/scissors instrument) to late dissection (tissue reflection showing the base of the tumor for assessment of deep margins) just before complete resection of the tumor.

**(A, B and C):** The upper panels and the illustration (i) show the focus on the instruments (since active surgery is going on) vs lower panels and illustration (ii) show purposeful change in the scope angle and the exposure of region of interest while keeping a perpendicular line angle of the camera which increases the fluorescence intensity of the tumor. **(C) Autofluorescence of the bipolar cautery** of the robotic arm used as **In-field reference** during intra-operative navigation. (C) fluorescent images show bipolar instrument and tumor (red and yellow arrows) autoscaling to brightest pixels (red and yellow arrow). (D fluorescent image on the last row also shows cautery induced artifacts (red arrow). **(D and E) Complete tumor resection and wound bed.** (D) Complete tumor resection done, the tumor tissue is released from all the margins, and the in-toto resection is imaged just before removal. (E) The margins are cleared of all the eschar, and the wound bed is irrigated to wash out the blood and debris before imaging.

**Supplemental Table 1. Firefly Modes on the da Vinci Xi**

| **FEATURES** | **STANDARD** | **SENSITIVE** |
| --- | --- | --- |
| **Primary Optimization** | Non-specific, blood-pool agents (ICG) | Tumor-targeted agents (panitumumab-IRDye800CW) |
| **Background Illumination** | Gray scale anatomy preserved | Visible background suppressed (darker field) |
| **Scope** | Compatible with the standard endoscope | Requires a dedicated sensitive endoscope not interchangeable with the Standard Firefly scope. |
| **Relative Sensitivity** | Inadequate for tumor-targeted agents | Enhanced sensitivity for detecting low-abundance fluorophores. |
| **Tools Availability** | Full | Limited (camera only) |
